# A novel *USH2A* variant in a patient with hearing loss and prenatal diagnosis of a familial fetus: a case report

**DOI:** 10.1186/s12920-021-01052-4

**Published:** 2021-08-10

**Authors:** Cong Zhou, Yuanyuan Xiao, Hanbing Xie, Shanling Liu, Jing Wang

**Affiliations:** 1 Department of Obstetrics and Gynecology, West China Second University Hospital, Sichuan University, Prenatal Diagnosis Center of Sichuan Province, 20 Section 3 Renmin South Road, Chengdu, 610041 Sichuan People’s Republic of China; 2 Key Laboratory of Birth Defects and Related Diseases of Women and Children, Ministry of Education, Sichuan University, Chengdu, People’s Republic of China

**Keywords:** Hearing loss, USH2A, Variant, Disease-targeted gene panel, Next generation sequencing, Prenatal diagnosis

## Abstract

**Background:**

Usher syndrome (USH) is the most common cause of inherited deaf-blindness. The current study aimed to identify pathogenic variants in a Chinese patient with hearing loss and to report the identification of a novel p.(Phe1583Leufs*10) variant in *USH2A,* which met the needs of prenatal diagnosis of the patient's mother*.*

**Case presentation:**

Genomic DNA obtained from a five-year-old girl with hearing loss was analyzed via the hearing loss-targeted gene panels. We identified the compound heterozygous variants c.8559-2A>G and c.4749delT in Usher syndrome type 2A (*USH2A*) gene as the underlying cause of the patient; the former variation has been reported in the literature, but not the latter. The parents of the girl were heterozygous carriers. The two variants were classified as pathogenic. Based on these findings, amniotic fluid samples were used for prenatal diagnosis of the couple's fetus, which was found to carry c.4749delT but not c.8559-2A>G variation. During the follow-up period of more than 9 months after the birth of the fetus, it was confirmed that the infant was healthy.

**Conclusions:**

The results of the present study identified two compound heterozygous *USH2A* variants in a patient with hearing loss and reported a novel *USH2A* variant which expands the spectrum of *USH2A* variants in USH.

**Supplementary Information:**

The online version contains supplementary material available at 10.1186/s12920-021-01052-4.

## Background

Usher syndrome (USH) is a clinically and genetically heterogeneous autosomal recessive disorder characterized by deafness and vision loss. Clinically, USH is divided into Type I (USH1), Type II (USH2), and Type III (USH3) [1]. The phenotypes of USH1 patients include congenital severe-to-profound deafness, vestibular areflexia, and onset of retinitis pigmentosa (RP) within the first decade of life; USH2 manifests as moderate-to-severe hearing loss, normal vestibular function, and onset of RP within the second decade of life; and USH3 is characterized by hearing loss, vestibular dysfunction, and onset of progressive, sporadic, and variable RP [2]. USH2 is the most common form of USH, and the Usher syndrome type 2A (*USH2A*) gene is thought to be involved in 74–90% of USH2 cases [3–5]. The *USH2A* gene was mapped to chromosome 1q41, with 72 exons and coding integral membrane protein usherin of 5202 amino acids (NM_206933.4). There are two isoforms of *USH2A*: a 170 kDa short isoform translated from 21 exons and a 580 kDa long isoform translated from an additional 51 exons [6].

In this study, two compound heterozygous pathogenic variants were identified in the proband, who presented bilateral moderate deafness. Based on the needs of the mother for prenatal diagnosis of deafness and the results of this study, we provide prenatal diagnosis for the fetus in this family. At the same time, we reviewed the relevant literature and explored the main clinical features, diagnosis, and treatment of USH2. The findings of this study can further enrich the database of pathogenic variation of the *USH2A* gene in the Chinese population and provide important information for carrier screening, molecular diagnosis, and prenatal diagnosis of USH2A patients.

## Case presentation

### Clinical findings

**Fig. 1:**
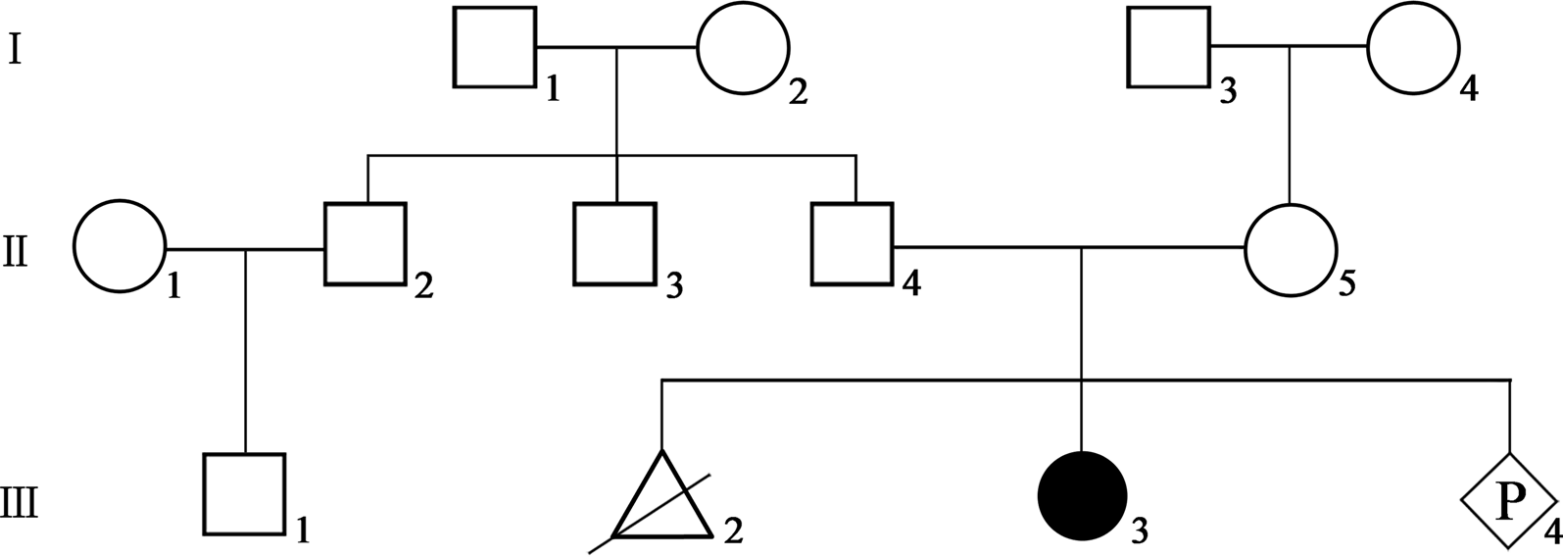
Pedigree of the patient’s family. There is only one affected individual. Males are represented by squares, females are represented by circles, and triangle refers to abortion in early pregnancy

A four-month pregnant, 35-year-old G3P1^+1^ Chinese woman came to the genetic consulting clinic of West China Second University Hospital of Sichuan University. The woman informed the doctor that she had an artificial abortion during her first pregnancy. She gave birth to a girl (the proband) at term following her second pregnancy; the child, who is now five years old, failed to pass otoacoustic emission bilaterally hearing screening at birth. We know from her medical records that she was examined in another hospital and diagnosed with moderate hearing loss in both ears (55 dB / 60 dB) more than one year ago; she subsequently began to wear hearing aids. The proband without absence of vestibular dysfunction, vision, or visual field involvement as her medical records. At present, the proband can communicate normally, without any other abnormal clinical manifestations. The parents of the girl are healthy and do not have a consanguineous marriage. The mother denied exposure to teratogenic environmental factors during pregnancy. The pedigree of this family is shown in Fig. 1. The woman asked for prenatal diagnosis of the fetus in the current pregnancy.

### Disease-targeted gene panel and Sanger sequence

Genomic DNA (gDNA) from the proband and her parents were extracted from leucocytes of peripheral blood samples using the QIAamp DNA Blood Mini Kit (Qiagen bioinformatics, Hilden, Germany) according to the manufacturer's standard extraction procedures. The quality and concentration of gDNA were assessed with the NanoDrop 1000 (Thermo Fisher Scientific, Wilimington, USA). For the proband, targeted gDNA was captured and enriched using two disease-targeted gene panels, the CM1132 Targeted Exome Capture Kit and M113 Mitochondrial Whole Gene Capture Kit (MyGenostics, Beijing, China), according to the manufacturer’s protocol. The CM1132 kit targeted 162 genes (Additional file 1: Table S1) known to cause deafness (include the genes’ whole exome, Coding + flanking intronic regions (10 bp) + deep intronic variants included in HGMD database), while the M113 kit (include the whole mitochondrial gene) contained two pathogenic hot-spot variants (NC_01292.1: m.1494C>T; NC_01292.1: m.1555A>G in MT-RNR1) that cause deafness. The captured and enriched gDNA libraries were sequenced using the NextSeq 500 platform (Illumina, California, USA) to generate paired-end reads for 150 cycles per reads. Adaptor, low quality, multiple N and short reads (< 40 bp) were removed by Cutadapt, and then the reads were mapped to the human genome reference (UCSCGRCh37/hg19) by the Burrows–Wheeler Aligner (BWA-MEM, version 0.7.10; https://www.plob.org/tag/bwa). Variants were called using the Genome Analysis Tool Kit (GaTK,version 4.0.8.1; https://www.broadinstitute.org/gatk/). annovar (version 1; http://annovar.openbioinformatics.org/en/latest/) was used to annotate the variants. After that, all the variants were filtered based on their frequency in public databases 1000 Genomes Project (http://browser.1000genomes.org), ExAC (http://exac.broadinstitute.org/), gnomad (http://gnomad.broadinstitute.org/), Esp6500.

(http://evs.gs.washington.edu/eVS) and human genome variant database (HGMD; https://portal.biobase-international.com/cgi-bin/portal/login.cgi). The variants with MAF < 0.05 were retained. Then, we applied several variant prediction tools including SIFT (http://provean.jcvi.org/), PolyPhen2 (http://genetics.bwh.harvard.edu/pph2/), MuatationTaster (http://www.mutationtaster.org/),GERP++ (http://mendel.stanford.edu/sidowlab/downloads/gerp/index.html) and SPIDEX (http://tools.genes.toronto.edu/), to predict the functional impact of candidate variants.

Finally, the pathogenicities of the detected sequence variations were analyzed according to the American College of Medical Genetics and Genomics (ACMG) guidelines [7] and Expert Specification of the ACMG/AMP Variant interpretation Guidelines for genetic Hearing Loss [8]. To confirm the variations found in the *USH2A* gene, Sanger sequencing was performed for the patient and her parents. The variation sites and amplification primers were as follows: c.8559-2 (forward 5′-GCCCAGAACTAAATGCCAGC-3′, reverse 5′-CCACGCATATATCACACGCA-3′) and c.4749 (forward 5′-CCTCAGTACCAGGCACCTAC-3′, reverse 5′-GCATTAAGGCCAGCTTTCGA-3′). The Sanger sequencing data were analyzed using Chromas software (http://technelysium.com.au/wp/chromas/).

### Prenatal diagnosis

Amniocentesis was performed at 20^+5^ weeks of gestation. Then, 20 ml amniotic fluid was collected, of which 16 ml was used for G-banding karyotype analysis (due to advanced maternal age) and the remaining 4 ml was used for prenatal diagnosis of the *USH2A* gene. The gDNA of amniotic fluid cells was extracted by DNeasy Blood and Tissue Kit (Qiagen bioinformatics, Hilden, Germany) [9]. First, the relationship between the family samples was confirmed by short tandem repeat analysis (DAAN gene, Guangzhou, China), which revealed that there was no maternal cell contamination in the amniotic fluid. Then, the nucleotide sequences of c.8559-2 and c.4749 position of *USH2A* genes in amniotic fluid cells were analyzed by Sanger sequencing.

### Genetic findings

**Fig. 2:**
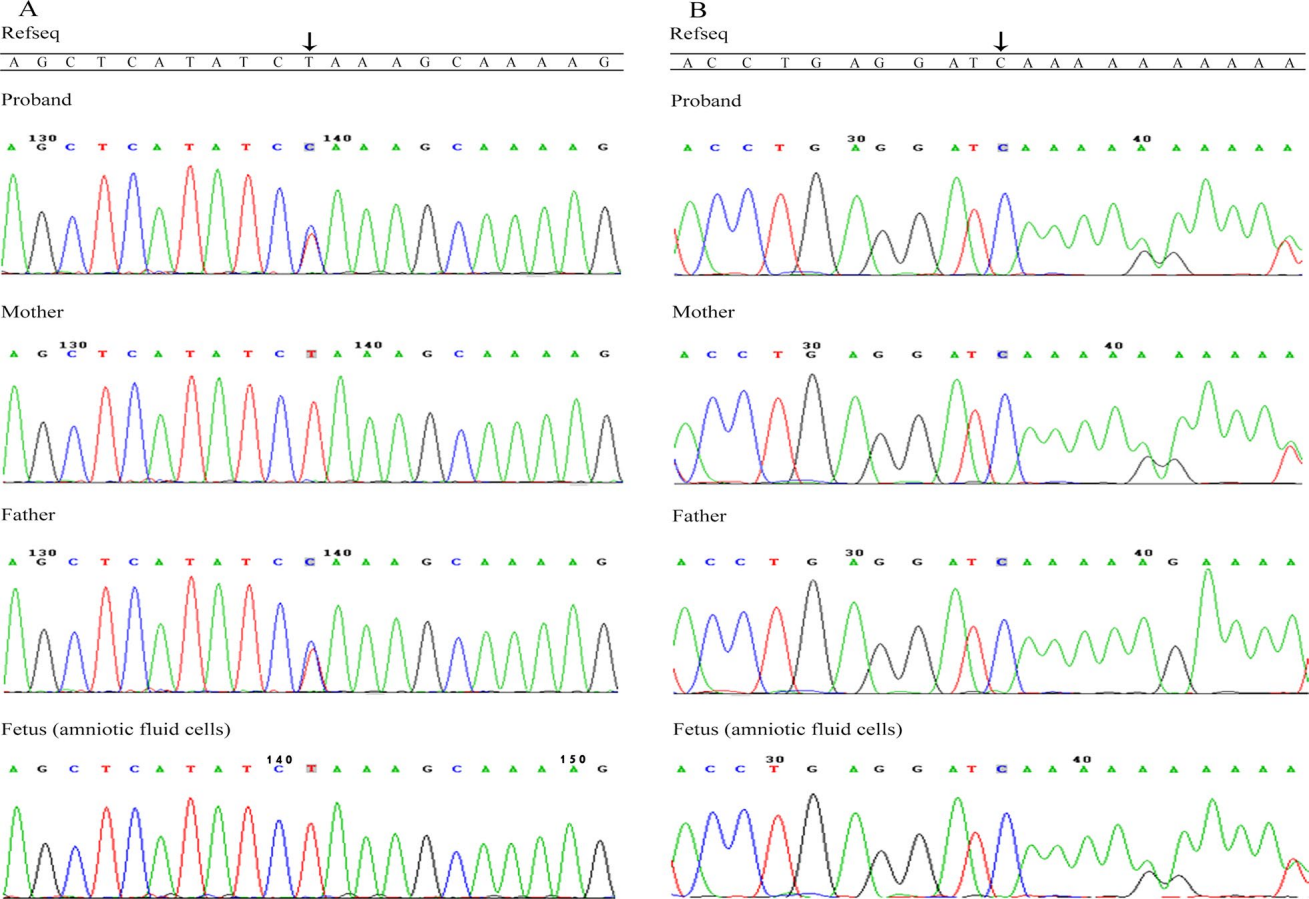
Sanger sequencing confirmation of the variants in *USH2A* identified in this study. **a** Sequences of the heterozygous splicing variant c.8559-2A>G and the corresponding wild-type sequence. **b** Sequences of the heterozygous frameshift variant c.4749delT (p.(Phe1583Leufs*10)) and the corresponding wild-type sequence

The average sequencing depth of the mitochondrial whole gene region was 4211X and no variant related to hearing loss was found in the M113 panel. With an average sequencing depth of 789X on the targeted regions, we identified 1610 genetic variants in the CM1132 panel. After extensive bioinformatics analysis, the two variants of the *USH2A* gene (c.4749delT in exon 22 and c.8559-2A>G in exon 43, NM_206933.4) of the proband needed further Sanger sequencing and family verification. Sanger sequencing of the family members showed that the proband was compound heterozygous, and that the parents were heterozygous carriers (Fig. 2a, b). The c.4749delT variant had not been reported in the general population databases and our results demonstrated that the deletion of the T nucleotides at exon 22 of *USH2A* is predicted to leads to a frameshift variant. This novel frameshift variant results in a premature stop-codon downstream of the variant p.(Phe1583Leufs*10), which has not been reported in the literature or databases. The frequency of c.8559-2A>G variant in gnomAD is ALL:0.0032%–EAS:0.043% (found in 8 individuals from East Asia). The variant is reported 10 times in the ClinVar (Variation ID:48604) database and published 16 times regarding to HGMD Professional 2020.4, several reports from China. Furthermore, RT-PCR analysis from a patient has been carrying out [10].

The result of the G-banding karyotype analysis of amniotic fluid cells showed 46, XY. The gene analysis of *USH2A* in amniotic fluid cells showed that there was a heterozygous variation (c.4749delT), but no variant at c.8559-2 (Fig. 2a, b). We followed up the fetus for 13 months; the mother of the proband delivered a baby boy with a height of 52 cm and weight of 4.4 kg at 40^+^ 1 weeks of gestation. The baby had normal hearing screening response after birth. He was checked regularly at the children's health care department and has not displayed any hearing problem or other abnormal phenotypes so far (9 months old).

## Discussion and conclusions

Usher syndrome is a severe disease resulting in significant vision and hearing impairments. Based on the phenotypic characterization, the disease has been classified into three subtypes [11]. USH2 patients show moderate-to-severe hearing loss, intact vestibular function, and onset of Retinitis pigmentosa (RP) within the second decade of life [12]. The patient in our study was five years old and exhibited bilateral moderate deafness, without absence of vestibular dysfunction, vision, or visual field involvement. Because RP in USH2 patients usually occurs after puberty, this proband cannot be excluded as USH2. However, there is considerable variability within the subtypes resulting in overlapping phenotypes between USH1, USH2, and USH3 [3–5]. Therefore, the diagnosis of USH in childhood may be difficult because although some features exist at birth, others appear as the child matures. Genetic testing should be undertaken when the clinical phenotype does not enable clear clinical diagnosis.

To date, sixteen genes have been reported to be associated with USH: nine are involved in USH1, three in USH2, two in USH3, and two are not specified. The *USH2A*, *USH2C* and *USH2D* genes are responsible for USH2 [13]. Due to the large number of coding exons existing in these genes, Sanger sequencing is not feasible for clinical application. In this study, we therefore attempted to target a group of genes responsible for hearing loss, including the *USH2A*, *USH2C,* and *USH2D* genes. As a result, we have proven that targeted deep exome sequencing of 162 known causative genes of hearing loss can serve as a fast and efficient way to diagnose USH2. Compared with whole exome sequencing and whole genome sequencing, the cost of the disease-targeted gene panel was much lower and the workload was lesser [14].

The *USH2A* gene, located on chromosome 1q41, consists of 72 exons. In mammalian photoreceptors, usherin is localized to a spatially restricted membrane microdomain at the apical inner segment recess that wraps around the connecting cilia, corresponding to the periciliary ridge complex described in amphibian photoreceptors [12]. *USH2A* gene variants have been implicated in the disease etiology of several inherited diseases, including USH2, nonsyndromic RP, and nonsyndromic deafness [15]. To date, 1348 variants in the *USH2A* gene have been reported in patients with USH2 or RP in the HGMD Professional 2020.4. Here, we reported two variants of *USH2A*, including one frameshift variant (c.4749delT, p.(Phe1583Leufs*10)) and one splicing variant (c.8559-2A>G),in the patient with hearing loss; the former variant was novel,while the later has been previously reported [16]. According to the criteria of ACMG and the Expert Specification of the ACMG/AMP Variant Interpretation Guidelines for Genetic Hearing Loss, the frameshift variant was pathogenic and the evidence include: (1) very strong evidence of pathogenicity (PVS1): frameshift; (2) Moderate evidence of pathogenicity (PM2): absent from controls in the general population databases; (3) Supporting evidence of pathogenicity (PP4): Patient's phenotype is highly specific for a disease with a single genetic etiology [7, 8]; the splicing variant was pathogenic and the evidence include: (1) very strong evidence of pathogenicity (PVS1): canonical splice site variants (− 2); (2) Strong evidence of pathogenicity (PS1): the same nucleotide in the splice consensus sequence as a known pathogenic variant; (3) Moderate evidence of pathogenicity (PM3): detected in trans with a pathogenic variant; (4)Supporting evidence of pathogenicity (PP4): Patient's phenotype is highly specific for a disease with a single genetic etiology [7, 8].

The variant c.8559-2A>G has been reported 10 times in the ClinVar database and published 16 times regarding to HGMD Professional 2020.4, several reports from China. The variant c.8559-2A>G in *USH2A* accounts for 19.1% of variants in a Chinese USH2 cohort [17, 18] and 26% in all Western Japanese USH patients. Furthermore, RT-PCR analysis from a patient has been carrying out [9]. Bioinformatic analysis predicted that the variant c.8559-2A>G would cause shearing abnormality, resulting in the skipping of exon 43 during transcription [2, 16, 19]. These results suggested that c.8559-2A>G may be one of the hot spot variants of the *USH2A* gene in Asian populations. Hence, variant screening for c.8559-2A>G in *USH2A* may prove very effective for the early diagnosis of USH2.

At present, the main abnormal phenotypes of USH are RP and sensorineural deafness. Despite the lack of cure for USH, cochlear implants can help improve the hearing functions of USH2 patients. Moreover, cochlear implants and sensory prosthesis implantation can improve the symptoms of hearing loss or retinal degeneration [2, 13]. At present, although there is no effective cure for human USH patients, virus vector and antisense oligonucleotide targeting therapies have been successfully used to treat USH in animal experiments [15].

In this report, we successfully performed genetic diagnosis of Usher syndrome by disease-targeted gene panel and have thus proven that this method can serve as a rapid, high-throughput, and efficient screening strategy. We describe a Chinese patient presenting clinical features compatible with USH2. Using a disease-targeted gene panel, we identified novel compound heterozygous variants in exons 22 and 43. The novel variant expands the spectrum of *USH2A* variants in USH. According to the criteria of ACMG, both variants were pathogenic. Specific DNA sequencing of the two variant sites was carried out in the fetus of the proband's mother. Subsequently, the mother of the proband gave birth to a healthy baby without any abnormal phenotype.

## Supplementary Information

**Additional file 1. Table S1**: 162 genes of the hearing loss-targeted gene panels.

## Data Availability

The raw sequence data reported in the paper have been deposited in the Genome Sequence Archive (Genomics, Proteomics & Bioinformatics 2017) in National Genomics Data Center (Nucleic Acid Res 2021), China National Center for Bioinformation/Beijing Institute of Genomics, Chinese Academy of Science, under accession number HRA001084 at https://ngdc.cncb.ac.cn/search/?dbId=hra&q=HRA001084. For the considerations about the security of human genetic resources and the confidentiality of participant, the data is not publicly available, but can be applied from the website or obtained from the corresponding author on reasonable request.

## Abbreviations

RP: Retinitis pigmentosa
USH: Usher syndrome
USH1: Usher syndrome Type I
USH2: Usher syndrome Type II
USH3: Usher syndrome Type III
*USH2A*: Usher syndrome type 2A
gDNA: Genomic DNA
ACMG: The American College of Medical Genetics and Genomics
DM: Disease variant
UCSC: The University of California Santa Cruz Genome Browser database
