# Supplementary material for: A novel USH2A variant in a patient with hearing loss and prenatal diagnosis of a familial fetus: a case report
Source: BMC Med Genomics. 2021 Aug 10;14:200. doi: 10.1186/s12920-021-01052-4 (PMC8353764; doi:10.1186/s12920-021-01052-4)
Supplement: Supplementary file 1 — Additional file 1. Table S1: 162 genes of the hearing loss-targeted gene panels. [file 12920_2021_1052_MOESM1_ESM.docx]

Table S1. 162 genes of the hearing loss-targeted gene panels.

| Gene_Symbol |  |  |  |  |  |  |
| --- | --- | --- | --- | --- | --- | --- |
| ACTG1 | COL9A2 | GATA3 | KRT9 | NEFL | PTPRQ | TIMM8A |
| ADGRV1 | CRYM | GIPC3 | LAMA3 | NELL2 | PTPRR | TJP2 |
| ALX3 | DFNA5 | GJB1 | LARS2 | NF2 | RDX | TMC1 |
| BSND | DFNB59 | GJB2 | LHFPL5 | OPA1 | RPGR | TMEM126A |
| CABP2 | DIABLO | GJB3 | LOXHD1 | OTOA | SALL1 | TMIE |
| CCDC50 | DIAPH1 | GJB6 | LRTOMT | OTOF | SALL4 | TMPRSS3 |
| CDH23 | DIAPH3 | GLYAT | MARVELD2 | OTOG | SEC23A | TMPRSS4 |
| CEACAM16 | DSPP | GPSM2 | miR-182 | OTOGL | SEMA3E | TNC |
| CHD7 | ECM1 | GRHL2 | miR-183 | P2RX2 | SERPINB6 | TPRN |
| CIB2 | EDN3 | GRXCR1 | MIR96 | PABPN1 | SIX1 | TRIOBP |
| CLDN14 | EDNRB | HARS | miR-96 | PAX3 | SIX5 | TRMU |
| CLPP | ELMOD3 | HARS2 | MITF | PCDH15 | SLC17A8 | TSPEAR |
| CLRN1 | ESPN | HGF | MPZ | PCDH9 | SLC19A2 | TYR |
| COCH | ESRRB | HMX1 | MSRB3 | PDZD7 | SLC26A4 | USH1C |
| COL11A1 | EYA1 | HOXA2 | MYH14 | PMP22 | SLC26A5 | USH1G |
| COL11A2 | EYA4 | HSD17B4 | MYH9 | PNPT1 | SMAD4 | USH2A |
| COL1A1 | FGF3 | IL13 | MYO15A | POLR1C | SMPX | WFS1 |
| COL1A2 | FGF8 | ILDR1 | MYO1A | POLR1D | SNAI2 | WHRN |
| COL2A1 | FGFR1 | KARS | MYO1E | POU3F4 | SOX10 |  |
| COL4A3 | FGFR3 | KCNE1 | MYO3A | POU4F3 | STRC |  |
| COL4A4 | FLNA | KCNJ10 | MYO6 | PROK2 | TBC1D24 |  |
| COL4A5 | FOXI1 | KCNQ1 | MYO7A | PROKR2 | TCIRG1 |  |
| COL4A6 | FREM1 | KCNQ4 | NDP | PRPS1 | TCOF1 |  |
| COL9A1 | FXN | KITLG | NDRG1 | PTPN11 | TECTA |  |
